# Effect of Nutrient Restriction and Re-Feeding on Calpain Family Genes in Skeletal Muscle of Channel Catfish (*Ictalurus punctatus*)

**DOI:** 10.1371/journal.pone.0059404

**Published:** 2013-03-19

**Authors:** Elena Preziosa, Shikai Liu, Genciana Terova, Xiaoyu Gao, Hong Liu, Huseyin Kucuktas, Jeffery Terhune, Zhanjiang Liu

**Affiliations:** 1 Fish Molecular Genetics and Biotechnology Laboratory, Department of Fisheries and Allied Aquacultures and Program of Cell and Molecular Biosciences, Aquatic Genomics Unit, Auburn University, Alabama, United States of America; 2 Department of Biotechnology and Life Sciences, University of Insubria, Varese, Italy; 3 Inter-University Centre for Research in Protein Biotechnologies “The Protein Factory”- Polytechnic University of Milan and University of Insubria, Varese, Italy; Temasek Life Sciences Laboratory, Singapore

## Abstract

**Background:**

Calpains, a superfamily of intracellular calcium-dependent cysteine proteases, are involved in the cytoskeletal remodeling and wasting of skeletal muscle. Calpains are generated as inactive proenzymes which are activated by N-terminal autolysis induced by calcium-ions.

**Methodology/Principal Findings:**

In this study, we characterized the full-length cDNA sequences of three calpain genes, *clpn1, clpn2, and clpn3* in channel catfish, and assessed the effect of nutrient restriction and subsequent re-feeding on the expression of these genes in skeletal muscle. The *clpn1* cDNA sequence encodes a protein of 704 amino acids, Clpn2 of 696 amino acids, and Clpn3 of 741 amino acids. Phylogenetic analysis of deduced amino acid sequences indicate that catfish Clpn1 and Clpn2 share a sequence similarity of 61%; catfish Clpn1 and Clpn3 of 48%, and Clpn2 and Clpn3 of only 45%. The domain structure architectures of all three calpain genes in channel catfish are similar to those of other vertebrates, further supported by strong bootstrap values during phylogenetic analyses. Starvation of channel catfish (average weight, 15–20 g) for 35 days influenced the expression of *clpn1* (2.3-fold decrease, P<0.05), *clpn2* (1.3-fold increase, P<0.05), and *clpn3* (13.0-fold decrease, P<0.05), whereas the subsequent refeeding did not change the expression of these genes as measured by quantitative real-time PCR analysis. Calpain catalytic activity in channel catfish skeletal muscle showed significant differences only during the starvation period, with a 1.2- and 1.4- fold increase (P<0.01) after 17 and 35 days of starvation, respectively.

**Conclusion/Significance:**

We have assessed that fasting and refeeding may provide a suitable experimental model to provide us insight into the role of calpains during fish muscle atrophy and how they respond to changes in nutrient supply.

## Introduction

Calpains constitute a superfamily of intracellular calcium-dependent cysteine proteases composed of one or two subunits. Tissue specific and ubiquitous calpain forms are known in organisms ranging from humans to microorganisms. Although many studies have focused on two mammalian isoforms, the µ- and m-calpains (known as Clpn1 and Clpn2, respectively), at least 15 different mRNAs or genes encoding polypeptides with sequence homology to the calpains have been identified in vertebrates [1]. The Clpn1 and Clpn2 isoforms share a 50–60% homology but differ in their *in vitro* sensitivity to calcium [2], [3]. Although these two isoforms are ubiquitously expressed in vertebrates, the expression of another family member of the calpains, calpain3 (Clpn3), is limited to skeletal muscle. The mammalian *Clpn3* gene is highly expressed in skeletal muscle where its mRNA levels are around 10-fold greater than those of *Clpn1* and *Clpn2* [4]. Furthermore, *Clpn3* differs from the first two isoforms in having alternative splicing domains. Not all calpain family members have been fully analyzed at the protein level [5], [6]. Calpains are heterodimers composed of a large 80 kDa catalytic subunit and a small 30 kDa regulatory subunit. The large subunit contains four domains (I to IV), whereas the small one contains two domains (V and VI) [7]. Domain I, a single α-helix composed of ten amino acid residues and located at the NH_2_-terminal region of the larger subunit, is very important for the stability and activation of some calpains. Domain II contains a highly conserved sequence of cysteine proteases, particularly around the amino acids forming the catalytic triad and it can be divided into two globular subdomains forming a catalytic cleft [8]. Domain III displays no apparent similarity to any other known protein sequences; thus it was not originally possible to assign its function. It is believed that it connects domain IV to domain II, forming a C2-like structure with an acidic loop that mediates Ca^2+^-dependent phospholipid binding and activation [1], [9]. Domain IV is a Ca^2+^-binding domain containing 5 EF-hands motifs, the classical helix-loop-helix structure found in calcium-binding protein families [10]. The 30-kDa regulatory subunit is identical in Clpn1 and Clpn2. Although this regulatory subunit is not essential for protease activity [11], it plays a role as a chaperone and is essential for proper conformation of the large subunit.

Clpn3 possesses the classical structure of the calpain family proteins including a proteolytic domain (domain II) and a calcium binding domain (domain IV) [12]. In addition to these structural domains, Clpn3 possesses three short Clpn3-specific sequences, known as NS, IS1, and IS2, located at the NH_2_-terminus within the protease domain, and between domains III and IV, respectively [1], [13]. These additional sequences are the primary reason for the larger molecular mass of Clpn3 (94 kD) with respect to that of the catalytic subunit of Clpn1 or Clpn2. Calpain3-specific amino acid sequences are thought to modulate calpain activity or to provide a nuclear localization signal. Using a yeast two-hybrid system, Sorimachi et al. [14] demonstrated that the IS2 sequence is capable of associating with the sarcomeric protein titin, possibly regulating Clpn3 distribution and activity. *Clpn3* is of major interest because the loss-of-function mutations in this gene in humans cause limb-girdle muscular dystrophy type 2A (LGMDA2) [15].

Calpain family proteins are generated in the cytosol as inactive proenzymes which translocate to membrane in response to increases in cellular levels of Ca^2+^. The initial effects of Ca^2+^ binding to calpain proenzyme include a conformational change that is essential to assemble the active site and some limited autolysis of the two subunits [11]. Many studies indicate that the calpain system has an important role both in muscle wasting associated with various types of muscular dystrophy, and in other conditions, such as burn injury, cancer, uremia and AIDS [16], [17], [18], [19], which are accompanied by loss of muscle mass, and in metabolic turnover of myofibrillar proteins [20]. Kumamoto et al. [21] suggested that calpains are involved in the early stages of muscle fiber degradation in muscular dystrophy. Indeed, several roles of calpains have been identified in muscle protein degradation involved in maintaining the myofibrillar structure. Salem et al. [22] demonstrated an increase in mRNA abundance of *clpn1* and *clpn2* in rainbow trout following food deprivation for 35 days, compared to fed controls. A marked increase in calpain mRNA during fasting was also reported in rabbits [23]. Little is known about the role of Clpn3 in muscle tissues. *clpn3* mRNA is transcribed at very high levels in skeletal muscle, but several studies have shown a decrease in *clpn3* mRNA copies in muscle wasting. It seems unlikely that Clpn3 has a degrading role in skeletal muscle; however, it may act as a signaling protease [20].

The calpain superfamily has received only limited attention in teleosts. Orthologues of mammalian *Clpn1* and *Clpn2* were described in *Oncorhynchus mykiss* [22], reporting similar sequence characteristics and domain structures. Presence of *clpn1* and *clpn2* genes has been reported in the zebrafish (*Danio rerio*) genome, too [24]. However, although certain components of the typical calpain system are clearly conserved in teleost fishes, the gene family is still poorly characterized [25]. Therefore, in this study, we set out to characterize three members of the calpain family in channel catfish *Ictalurus punctatus* which is a major aquaculture species in the United States [26]. The full-length nucleotide sequences of the channel catfish *clpn1*, *clpn2* and *clpn3*, were determined. The evolutionary relationships between channel catfish calpain genes and their orthologs from a broad range of vertebrate species were deduced by *in silico* phylogenetic analyses. In light of the literature, we quantified the mRNA abundance of *clpn1*, *clpn2, clpn3, and titin* genes in the skeletal muscle of channel catfish in response to fasting and re-feeding. We have also attempted to assess the involvement of calpain family genes in skeletal muscle atrophy by characterizing the time course of calpain enzyme activity, assuming that fasting may provide a suitable experimental model to provide us insight into the role of calpains during muscle atrophy and how they respond to changes in nutrient supply.

## Materials and Methods

### Ethics Statement

All experimental procedures described in this study followed the protocol PRN-2010-1816 approved by the Institutional Animal Care and Use Committee (IACUC) of Auburn University. No other license was required for this study.

### Identification of *clpn1*, *clpn2*, *clpn3* genes in Catfish

To identify the genes considered in this study, RNA-Seq assembly from previous work [27], [28] and the catfish whole genome sequence assembly (unpublished data) were searched against using available zebrafish (*Danio rerio*) genes as queries. Contig sequences were further manually screened for full-length sequences using BLASTX alignments by searching against the *nr* database. A full-length cDNA was defined as a sequence with a conserved start codon, a complete open-reading frame (ORF), and a stop codon using ORF Finder (http://www.ncbi.nlm.nih.gov/projects/gorf/). The predicted ORFs were verified by BLASTP against the NCBI non-redundant protein sequence database. The conserved domains based on sequence homology were identified by the Simple Modular Architecture Research Tool (SMART) (http://smart.embl-heidelberg.de) and further confirmed by predicting the conserved domain using BLAST. In order to predict whether the calpains are cytoplasmic, the PSORT program was used (http://psort.nibb.ac.jp/form2.html). Proteolytic cleavage sites were predicted with the Epestfind program (http://mobyle.pasteur.fr/cgi-bin/portal.py?#forms::epestfind).

A phylogenetic tree was constructed using the Molecular Evolutionary Genetics Analysis (MEGA 4.0) software [29]. The amino acid sequences of calpains, from various species retrieved from GeneBank, were aligned by ClustalW and then a neighbor-joining tree was constructed. Data were analyzed using Poisson distance correction and gaps were removed by deleting them completely. The topological stability of the trees was evaluated by 10,000 bootstrap replications.

### Animals and Feeding Protocol

One hundred fifty channel catfish (*Ictalurus punctatus*) fingerlings from a single artificial spawn (full siblings), of 17.87±5.6 g mean weight, age 12 months, were obtained from the Hatchery of the Department of Fisheries and Allied Aquacultures, Auburn University. Fish were reared at the Hatchery in a 1 m^3^ fiberglass tank connected to a flow-through system with a flow-rate of 4 L/min. There was no fish loss during the experiment.

Other water parameters were strictly controlled: temperature 26±1.5, pH 7.9±0.49, total ammonia 0.6 mg/L, and nitrite below 0.16 mg/L. Dissolved oxygen was maintained at over 99% of the saturation value. The water quality parameters were kept constantly in manual mode in each tank throughout the study.

Fish were randomly divided into six experimental groups (30 fish each) and were let to acclimate for 15 days before starting the experiment. Each group was assigned to a net pen (30×30×60 cm) collected to the same water recycling system.

**Figure 1:**
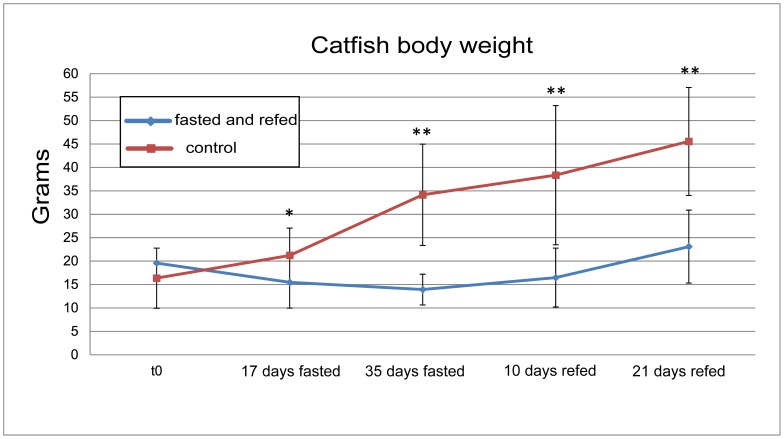
Fish body weight during the experiment. The means of fifteen animals at each time point are shown. Bars indicate standard deviation. Differences were determined by Student’s t-test. Asterisks indicate significantly different means between “control” and “fasted and refed” within each time point (*P<0.05; **P<0.01).

Fish in these three groups were manually fed to apparent satiety (fed control) a commercial fish diet (Zeigler Bros: Finfish hi-performance slow sinking; Gardeners, PA) containing: 42% protein, 16% fat, 3.5% fiber and 1.1 phosphorus, whereas fish of the other three groups were deprived of food for 35 days and then re-fed to apparent satiety for 21 days with the same type of feed utilized before fasting. This starvation regimen was based on the study of Tripathi et al. [30] who showed that the maximum effect of fasting on catfish muscle protein content occurs after 35 days of starvation. At the beginning of the experiment, the mean body weight of the catfish was 16.36±6.42 g for the control group and 19.58±4.32 g for the “food-deprived” group. Five fish from each experimental group were randomly sampled at the following time points: 17 days after fasting (17 days fasted), at the end of fasting period (35 days fasted), and then sequentially at 10 (10 days refed) and 21 days following re-feeding (21days refed). The biometric data of the animals sampled during the trial, are reported in Table 1. Fish weight at each sampling point is shown in Figure 1. Fish were anesthetized by tricaine methanelsulfonate (MS222; Argent Chemical Laboratories) exposure at 300 mg/L before sampling, and then the body weight, total and standard length were measured. White muscle was dissected from each sampled fish, frozen immediately in liquid nitrogen, and then stored at -80°C until the moment of molecular biology analysis.

**Table 1:** Mean of total length (TL), standard length (ST) and Weight (W) for each group of samples animals.

	Control group			Food-deprived group		
Sampled group	TL (cm)	SL (cm)	W (g)	TL (cm)	SL (cm)	W (g)
**To**	11.6±1.7	9.5±0.79	16.35±6.42	12±1.5	9.03±1.27	19.58±4.32
**17d**	12.5±1.27	9.5±0.94	21.22±5.82	12.4±1.82	9.5±1.12	15.46±5.48
**35d**	14.65±1.24	11.97±1.23	34.15±10.81	11.70±1.41	9.49±0.68	13.92±3.27
**10d**	15.07±2.13	12.8±1.69	38.36±15.86	12.5±1.2	10.4±0.95	16.49±6.28
**21d**	16.35±1.54	13.53±1.22	45.56±11.56	13.7±1.77	11.07±1.4	23.09±7.78

### Quantitative Real-time RT-PCR

Quantitative real-time PCR (qRT-PCR) was used to quantify expression levels of *clpn1*, *clpn2*, *clpn3*, and titin genes in channel catfish. Total RNA was isolated from the muscle of five fish from each of the experimental group collected at four time points, as described above. A pooled sample consisted of RNA extracted from the muscle tissue of five fish. We pooled the RNA samples in order to increase the number of analyzed animals because we are more interested in the average gene expression under the treatment than in variations among individuals; in this way 15 fish (3 pools of 5 fish each) were analyzed for every time point of sampling. For RNA extraction, white muscle samples were homogenized using a mortar and pestle under liquid nitrogen and then TRIzol reagent (Invitrogen) was used following the manufacturer’s protocol. The concentration of total RNA was quantified using a spectrophotometer (Ultrospec 1100 pro, Amersham Bioscience, Piscataway, NJ). One microgram of each RNA sample was then reverse transcribed to cDNA using the iScript cDNA Synthesis kit (Bio-Rad, Hercules, CA). The qRT-PCR reactions were performed in triplicate on a CFX96™ Real Time PCR Detection System (Bio-Rad, Hercules, CA) using a SsoFast EvaGreen Supermix (Bio-Rad, Hercules, CA) following the manufacturer’s protocol with a modification, only 100 ng of total RNA was used in one reaction. Oligonucleotide primers for calpains were designed from the most diverse sequence regions and could amplify only their respective gene. The specificity of PCR products were confirmed by size matching to predicted amplification products, as well as by specific melting curve analysis of realtime PCR, using Bio-Rad CFX manager software. Briefly, all real time RT-PCR reactions were performed in a 10-µl total reaction volume (9 µl master mix and 1 µl RNA template). A five-step experiment protocol was run on the CFX96™ Real Time PCR Detection System: (i) reverse transcription, 20 min at 61°C; (ii) denaturation, 30 s at 95°C; (iii) amplification repeated 50 times, 1 s at 95°C, 1 s at 55°C, 13 s at 72°C; (iv) melting curve analysis, 5 s at 95°C, 15 sec at 65°C, then up to 95°C at a rate of 0.1°C per second; (v) cooling, 30 s at 40°C. Concentration of cDNA in each sample was calculated from the standard curve. Beta-actin gene, a single copy gene in the genome, was used as an internal control as it was found to be stably expressed with fasting treatment [31], and we have used beta-actin gene as internal control of gene expression in catfish for numerous gene expression studies [32], [33], [34], [35]. The primers used in qRT-PCR reactions are listed in Table S1.

### Calpain Enzymatic Activity Assay

Muscle tissues from the same individuals as those used for the transcriptomic analysis were used for this assay. Calpain catalytic activity in channel catfish muscle tissues was measured at 17 and 35 days of fasting, and 10 and 21 days of refeeding. Activity was expressed as fluorescence units per milligram of tissue that increased the fluorescence during the 30-min incubation period (P<0.01). Calpain enzyme activity was measured using the InnoZyme™ Calpain 1/2 Activity Assay Kit according to the manufacturer’s protocol (Merck KGaA, Darmstadt, Germany). Twenty milligrams of each muscle sample was homogenized in a CytoBuster™ Protein Extraction Reagent (Merck KGaA, Darmstadt, Germany); the concentration was measured with a BCA™ protein Assay kit (Merck KGaA, Darmstadt, Germany); and the lysate was diluted to a final concentration of 200 µg/ml. After combining 100 µl reaction buffer and 50 µl of calpain substrate, the reaction was incubated at room temperature for 30 min in the dark with gentle shaking. The calpain activity, expressed in relative fluorescence units (RFU), was measured at room temperature as a function of time (30 min) using a Synergy HT Multi-Mode Microplate Reader (BioTek) by reading fluorescence values at an excitation wavelength of 320 nm and an emission wavelength of 480 nm.

### Statistical Analysis

Statistical analysis of the mRNA abundance of target genes was performed using a software tool, Relative Expression Software Tool 384 v.1 (REST©), assuming 100% amplification efficiencies [36], [61]. This software permits the comparison of numerous genes by using a common reference gene, while taking into account differences in reaction efficiency that are not addressed by the commonly used ΔΔCt method, with the ratio = (Etarget)▵CP target (control−treated)/(Eref)▵CP ref (control−treated). REST© employs a non-parametric method to assess the statistical significance of any differences between groups, calculating P values on the basis of the pairwise fixed reallocation randomization test which jointly reallocates the Ct values for reference and target genes to control and sample groups, and then calculates the resulting expression ratios on the basis of the mean values.

The expression ratio results were then tested for significance (p<0.05) by a Pair Wise Fixed Reallocation Randomization Test©. In the current study data were normalized against β-actin gene expression levels and calculated using 10 000 randomizations.

The activity values of calpain enzyme were analyzed by one-way ANOVA, with a level of significance set at p<0.05. Where variance proved to be significant, differences were evaluated using Duncan’s test.

The statistical analysis of fish biometric data were performed with the Student t test with a significance level set at p<0.05 or p<0.01.

## Results

### Sequence Determination of Full-length *clpn1*, *clpn2* and *clpn3* cDNAs in Catfish

**Figure 2:**
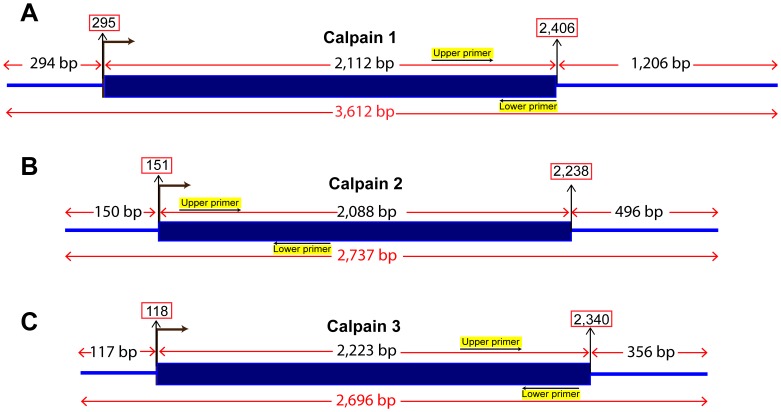
Schematic diagram of the full length cDNAs of channel catfish calpain catalytic subunits: (A) *clpn1*; (B) *clpn2*; (C) *clpn3*. The positions of the primer sets used for real-time RT-PCR transcript quantification of each gene are indicated with the respective sequences.

The cDNA sequences of the three genes in this work were obtained from our assembled full-length cDNAs generated from RNA-Seq assembly of a doubled haploid channel catfish [27]. The use of a doubled haploid fish, which harbors two sets of identical chromosomes, ensured to get the accurate sequences. Furthermore, the cDNA sequences were confirmed by comparison with the catfish whole genome assembly (unpublished data) which was also originated from sequencing a doubled haploid channel catfish. All full-length sequences were deposited in GenBank with continuous accession numbers of JQ60926-JQ60928. The information for full-length cDNAs of channel catfish *clpn1, clpn2, and clpn3* is summarized in Figure 2.

Amino acid sequence analysis of Clpn1, Clpn2 and Clpn3 revealed the characteristic domains of the calpain catalytic subunits: I (pro peptide), II (cysteine catalytic site), III (“electrostatic switch”), and IV (five Ca^2+^-binding EF-hands). The catalytic triad, C (cysteine), H (histidine), and N (asparagine) (Figure S1 and Figure S2), together with the surrounding sequences were found to be conserved in Clpn1 and Clpn2; however, an asparagine residue is not conserved in Clpn3 (Figure S3). The overall amino acid sequences of the catfish Clpn1, Clpn2, and Clpn3 are highly conserved as compared with those of Atlantic salmon, rainbow trout, zebrafish, and Atlantic halibut (Figures S1, S2, S3).

### Phylogenetic Analysis of Calpains

**Figure 3:**
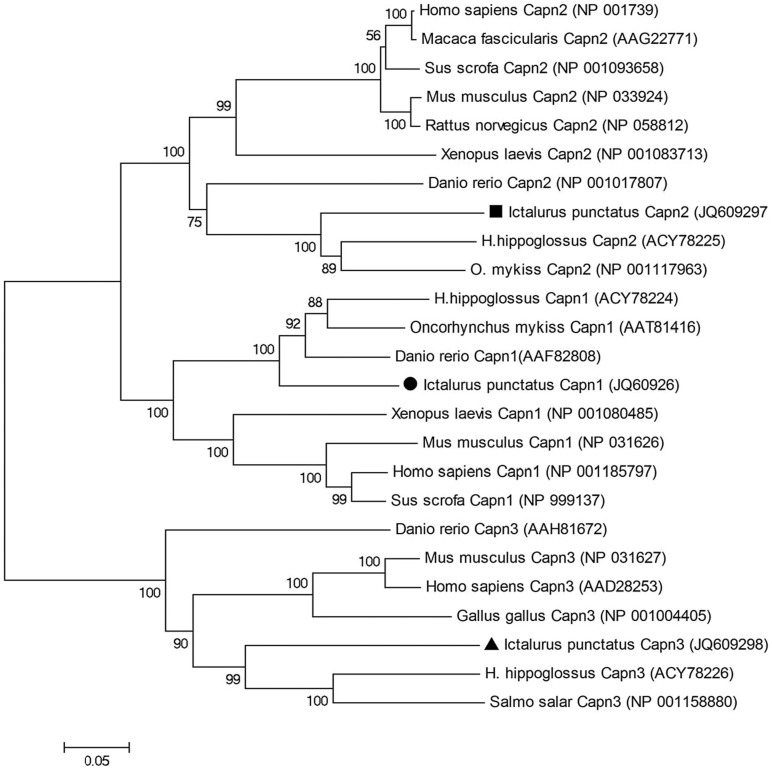
Phylogenetic tree comparing the amino acid sequences of Clpn1, Clpn2, and Clpn3 in catfish with those of other vertebrate species. GenBank accession numbers of each sequence used in the analysis are indicated on the right side of the species name. The phylogenetic tree is generated based on ClustalW generated multiple sequence alignment using the neighbor-joining method which ignores gaps as implemented in MEGA 4.0. The topological stability of the neighbor tree was evaluated by 10,000 bootstrapping replications, and the bootstrapping percentage values are indicated by the numbers at the tree nodes.

A phylogenetic analysis was conducted based on the amino acid sequences of Clpn1, Clpn2 and Clpn3, including 25 sequences from a total of 12 taxa. As one can see in Figure 3, the phylogenetic tree showed two main clades. An expected vertebrate-wide sub-branching pattern inherited from a common vertebrate ancestor within each calpain family member branch, was observed. The phylogenetic tree showed high confidence level for branching order of the majority of organisms. A total of 13 out of 22 nodes had values of 100 out of 100 replicates (Figure 3). In this tree, deduced amino acid sequences could be grouped into two main clusters with high bootstrapping values comprised of Clpn1, Clpn2 and Clpn3. Similarly, Clpn1 and Clpn2 sequences formed two separate clusters with high bootstrapping values. Both Clpn1 and Clpn2 sequences from teleost fish consistently grouped together with moderate bootstrap support, suggesting orthologous relationship between these proteins.

### Expression of Calpain and Titin Genes in Skeletal Muscle during Fasting and Re-feeding

**Figure 4:**
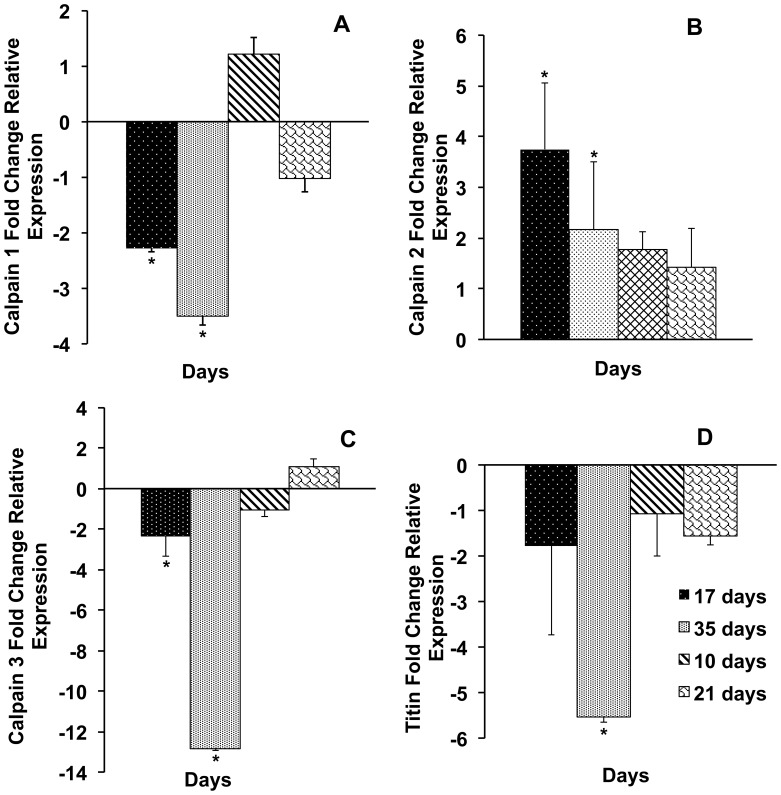
Effect of starvation and re-feeding on catfish muscle *clpn1*, *clpn2*, *clpn3*, and titin genes expression. Real-Time RT-PCR analysis of catfish (*Ictalurus punctatus*) *clpn1, clpn2, clpn3*, and titin transcripts abundance in the muscle after 17 and 35 days of starvation and then after 10 and 21 days of re-feeding. Fold change indicates the ratio of gene expression, all compared with t0, and normalized using an internal reference gene (catfish β-actin). The statistical analysis was conducted using the software REST. The bars indicate mean expression of 3 tested pools of 5 fish each ±SD. Asterisks indicate significant difference (P<0.05).

qRT-PCR was used to quantify the expression of *clpn1*, *clpn2, clpn3* and *titin* genes in skeletal muscle of catfish. The effects of fasting and re-feeding on the expression of these genes are shown in Figure 4. Comparison of channel catfish *clpn1* and *clpn3* gene expressions (Figure 4A, C) from the control and the treatment group (deprived of food for 35 days and subsequently refed for 21days) showed that the transcript levels were significantly down-regulated (2.3- to 13.0- fold, P<0.05) at days 17 and 35, whereas the expression remained unchanged at day 10 and 21 of the re-feeding phase. The expression of *clpn2* gene (Figure 4B) appeared to be up-regulated throughout the experimental period. Although this up-regulation persisted during the entire trial, it was significant only during the fasting period: 3.8-fold increase (P<0.05) at 17 day fasted, and 2.2–fold increase (P<0.05) at 35 day fasted (Figure 4B).

With regard to titin, comparison of channel catfish gene expressions from the control and the treatment group showed that the mRNA levels were significantly down-regulated (5.5-fold, P<0.05) at day 35 of fasting phase, whereas the expression remained unchanged at day 17 of fasting, and at days 10 and 21 of the re-feeding phase (Figure 4D).

### Calpain Enzymatic Activity

**Figure 5:**
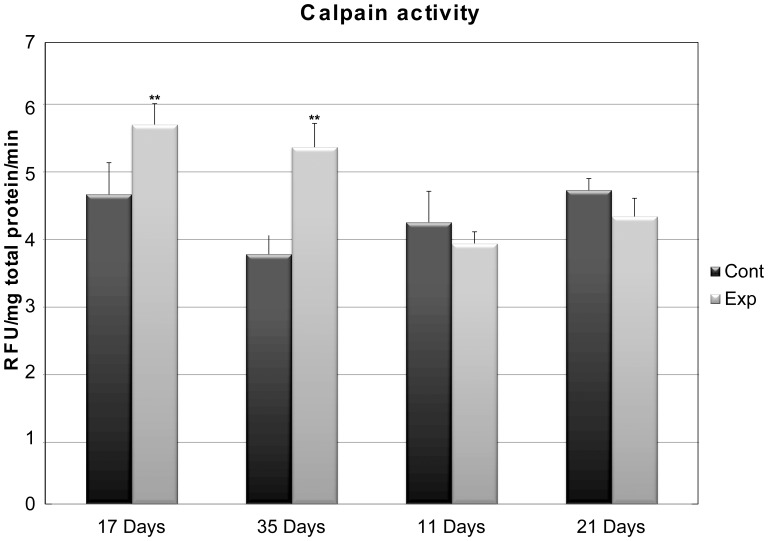
Change in calpain catalytic activity in catfish muscle tissue. Calpain activity was expressed as relative fluorescence unit for mg of tissue where fluorescence increased during 30 min incubation period. Double asterisks indicate highly significant difference between the experimental and control group at the same time (P<0.01).

Calpain catalytic activity was increased in fasting channel catfish, 1.2-to 1.4–fold at 17 and 35 days of fasting (Figure 5), whereas no significant changes were observed during the period of re-feeding. The relative increase in calpain catalytic activity after 17 days of fasting was considerably higher than that of the remaining time points, indicating increased *in situ* proteolytic activity.

## Discussion

Calpain family proteins present in the cytoplasm as pro enzymes have an important role in muscle metabolism. Members of this family of proteins actively translocate to cellular membranes in response to increases in cellular Ca^2+^ levels. Several studies showed that these proteins are directly or indirectly involved in differentiation, atrophy, and regeneration of muscle and it is evident that they are involved to a major degree in certain types of muscular dystrophy [37], [38], [39]. In this study, we identified the full-length nucleotide sequences of the channel catfish calpain catalytic subunits *clpn1*, *clpn2* and *clpn3*, and the partial sequence of titin, analysed their expression levels, and measured the catalytic activity of calpain in skeletal muscle of channel catfish during fasting and re-feeding. Deduced channel catfish Clpn1, Clpn2, and Clpn3 amino acid sequences contained four conserved characteristic domains seen also in other organisms for which sequence information is available. The catalytic triad C, N, and H in domain II is conserved in both catfish Clpn1 and Clpn2 (Figure S1 and Figure S2); however, the asparagine residue in the Clpn3 was not conserved in our species (Figure S3), confirming a role of Clpn3 as a signaling protease rather than it having in general, a degrading role [20]. Homologous comparison of the various domains of catfish calpain family genes showed that domains II and III were the most conserved, and catalytic domain was likely subjected to higher selective pressure during evolution [22]. On the other hand, the other domains had rather divergent sequences, probably because of their different physiological functions.

Phylogenetic analysis based on the amino acid sequences of Clpn1, Clpn2, and Clpn3 revealed two main clusters that include these members of the calpain family (Figure 3). However, Clpn1 and Clpn2 sequences further branched out within their main cluster. Furthermore, Clpn1, Clpn2 and Clpn3 were separated, delineating the mammalian and teleost calpain protein families. It has been hypothesized that a series of possible gene duplication events may explain the closer evolutionary relationships among the main components of the calpain family proteins [40].

Many studies indicate that the calpain system has an important role both in muscle wasting associated with various types of muscular dystrophy and in other conditions accompanied by loss of muscle mass and metabolic turnover of myofibrillar proteins [20], [41]. In addition, it has been suggested that calpain does not play a direct role in protein degradation at the amino acid level, but rather it disrupts normal functioning of the myofibrils by cleaving titin or nebulin [7], allowing thus further protein breakdown into amino acids via the ubiquitin-proteasome pathway [42]. In our study, analysis of catfish calpain activity showed a 1.2- and 1.4-fold increase at 17 and 35 days of fasting, respectively (P<0.01) (Figure 5). These results are consistent with the increase in calpain activity observed in other teleosts during a fasting period [22]. Similar results were observed also in mice where enzyme activity increased 2.9-fold during fasting and decreased on re-feeding [42], indicating a role for calpain during extended starvation. Du et al. [43] observed a decrease in calpain activity during the period of re-feeding, in cows. Calpain activity remained elevated during the fasting period in channel catfish muscle tissue compared to the control group.

As emphasized by Bartoli and Richard [44], the relative contribution of each calpain in the development of muscle atrophy is difficult to assess, and the increased mRNA and/or protein levels do not necessarily reflect increased calpain activity [45]. Less than 50% of the enzymes in cells have their activity regulated at the transcriptional level. Both the calpains are phosphorylated, and phosphorylation affects their activity [20]. Measurements of protein levels and enzyme activities are more directly related to intracellular enzyme activity than message levels [20]. Their activity, as assessed by spectrofluorimetric assays with fluorescent substrates, is increased during disuse, fasting, and remodeling induced by chronic low-frequency stimulation [46], [47], [48], [50], [53].

Expression analysis of calpain family genes in channel catfish muscle tissue during fasting showed a significant increase in the level of transcripts encoding *clpn2*, whereas the transcript levels of *clpn1* were, during the same period, decreased (Figure 4A, B). These results are consistent with those shown in lambs [49], [50]. Furthermore, the expression levels of *clpn2* but not those of *clpn1* in fasted catfish (Figure 4) are consistent with the results obtained by Salem et al [22] who found a 2.2 and 6.0-fold increase in mRNA abundance of *clpn1* and *clpn2*-like, respectively, in skeletal muscles of fasted versus fed rainbow trout. However, in another study by MacQueen et al [25] it was shown that *clpn1*, but not *clpn2*-like was transcriptionally upregulated in the fast-twitch skeletal muscle of fasted halibut compared to an extended period of subsequent recovery feeding associated with muscle anabolism. This provides some evidence that halibut *clpn1* is transcriptionally downregulated during the switch from catabolism to anabolism in skeletal muscle [25].

It was a surprised observation that catfish *clpn2* was upregulated during the refeeding period whereas *clpn1*, *clpn3*, and titin were all down-regulated (Figure 4). At present it is not clear for us the physiological mechanism involved in this differential regulation of the *clpn2*. However, a potential explanation for this increase in transcripts abundance might also reside in a higher physiological demand of Clpn2 protein. It should be taken into account, that calpains are regulatory proteases, so they are involved in a plenty of intracellular processes, controlled by calcium-ions. Different substrates are cleaved by calpains at different positions. Nowadays more than one hundred proteins such as cytoskeletal substrates, kinases and phosphatases, integral membrane proteins, receptors, ion channels, and transcription factors are proven to be substrates of calpains. Furthermore, in the study by MacQueen et al [25], halibut *clpn3* showed the same pattern of expression of *clpn2* gene in our experiment. Indeed, *clpn3* was expressed at its lowest levels in halibut fasted skeletal muscle and was strongly upregulated during refeeding when an increase in skeletal muscle mass was observed. This has provided the first evidence for a pro-myogenic role for teleost calpain-3, potentially in relation to sarcomere formation during muscle growth.

Unlike differences found in the levels of calpains transcripts, no significant changes were observed in total calpain catalytic activity during the recovery feeding of catfish (Figure 5). It is well documented that Clpn1 and Clpn2 have different i*n vitro* calcium sensibility, in the range of 15–50 uM and 250–1000 uM, respectively [3]. Increased level of intracellular calcium determines an enhanced sensibility of calpain for Ca2+ through association with phospholipid structure is generally identified as the primary mechanism structures of calpain activation in vitro [51]. *In vivo*, a translocation of calpain activities from cytosolic to particulate (i.e, membrane, and protein associated) structures has been shown to be a physiologically relevant event for calpain activation [52], [53]. Indeed, it was demonstrated that calpain 1 changes its localization when [Ca2+] increases and autolysis occurs [54]. The authors suggested that this regulation could occur *in vivo* in situations where [Ca2+] is increased, such as eccentric exercise and Duchenne’s muscular dystrophy [54].

Different studies have shown that the increase of the degradation products of the filament of muscle and calpain 1 autolysis in the soleus muscle may be involved in remodeling of the cytoskeleton in the early stages of muscle wasting [7], [42], [55]. Furthermore, in a study on the rat soleus muscle [37], the calpain2 autolysis which is acknowledged as an indicator of calpain activity was increased in the soluble fraction of immobilized soleus muscles. Thus the increased calpain 2 autolysis suggested that calpain 2 was more active in this fraction after immobilization, which may imply that calpain 2 plays a role in the signaling pathways that induce muscle atrophy. Indeed, the limited cleavage sites on substrate proteins suggest that calpains have a signaling or regulatory function rather than a digestive function, such as the proteasome has [37]. Interestingly, the data in this case showed that the increase of autolyzed calpain 1 in the particulate fraction was accompanied by a reduction in the 80-kDa form, while total calpain 1 content did not change, after 5 days of immobilization. Conversely, the increase in calpain 2 autolysis in the soluble fraction was not accompanied by a change in 80-kDa form content. Thus total calpain 2 content increased after immobilization in the soleus soluble fraction, and it can be assumed that calpain 2 was upregulated during this short period of disuse, whereas calpain 1 was not [37].

There has been a great deal of interest in understanding the role of Clpn3 because it was shown that the disruption of this gene causes limb-girdle muscular dystrophy type 2A (LGMD2A) in humans [15]. In our study, results show that the expression of *clpn3* is decreased with fasting; this is in contrast to the roles of Clpn1 and Clpn2, which positively regulate the cellular events leading to muscle atrophy [44]. Similar results have been obtained in previous studies [56] in which a decrease in *clpn3* gene expression in skeletal muscle of interleukin-6 (IL-6) was observed in transgenic mice suffering from atrophy of both red and white muscles. Clpn3 acts as a signaling protease, but it appears unlikely that it has in general a degrading role in skeletal muscle [20]. Further studies have determined that Clpn3 may participate in sarcomere formation or maintenance through its interaction with titin, a giant protein that serves as a molecular ruler of the sarcomere, orchestrating alignment of sarcomeric proteins during development and in postnatal muscle [57]. Interestingly, in our study, the recovery phase subsequent to fasting is associated with an increase in *clpn3* expression (Figure 4C). Models developed to test hindlimb muscle remodeling in the mouse suggested that, in addition to sarcomere remodeling, Clpn3 also promotes myofibrillar protein in postnatal muscle [58].

It is well established that muscle atrophy involves primarily the loss of intracellular proteins; however, only little attention has been directed towards possible changes in the production of extracellular matrix components during muscle atrophy [59]. We analyzed the expression levels of titin in channel catfish during the course of the experiment to determine if any interaction between calpain and titin can be deduced from the expression patterns. Molecular interaction between titin and calpain, either by direct binding or through an intermediate protein, myospyrin, was attributed to a protective mechanism by which these interactions would protect Clpn3 from autolysis [60]. Down-regulation of both titin and Clpn3 in catfish throughout the starvation period in our experiment may be indicative of these interactions; however, further studies are needed to examine the true nature of these events.

In conclusion, we have identified and characterized the full-length cDNAs of *clpn1*, *clpn2*, and *clpn3* genes in channel catfish. The architectures of these genes are similar to those of the other homologous calpain family members from a wide range of organisms. After a starvation and re-feeding period in a preliminary step in our study, the expression patterns of calpain genes and the catalytic activity of the protein indicated either further degradation of myofibrillar proteins for amino acid supply or the qualitative remodeling of the muscle. Moreover, under wasting conditions, *clpn3* was immediately down-regulated, suggesting that its activity is different to that of the ubiquitous calpains required for skeletal muscle homeostasis.

## Supporting Information

Figure S1 **Comparison of the deduced amino acid sequences of calpain 1 orthologs in catfish and other organisms.** Comparison of the deduced amino acid sequences of catfish calpain 1 with homologous sequences of *Danio rerio* (Dre, GenBank, AAF82808), *Oncorhynchus mykiss* (Omy, GenBank, AAT81416), and *Hippoglossus hippoglossus* (Hhi, GenBank, ACY78224). Identical amino acid residues are highlighted. The four catalytic subunits: I (pro-peptide), II (Cysteine catalytic site), III (“electrostatic switch”), and IV (five Ca^2+^-binding EF-hands), are shown. The catalytic triad residues are boxed, highlighted in green and marked with a star underneath (C: cysteine; H: histidine, N: asparagine).
(DOCX)

Figure S2 **Comparison of the deduced amino acid sequences of Clpn2 of catfish and other organisms.** Comparison of the deduced amino acid sequences of catfish Clpn2 with homologous sequences of *Danio rerio* (GenBank, NP_001017807), *Oncorhynchus mykiss* (GenBank, NP001117963) and *Hippoglossus hippoglossus* (GenBank, ACY78225). Identical amino acid residues have been highlighted. The four catalytic subunits: I (pro-peptide), II (Cysteine catalytic site), III (“electrostatic switch”), and IV (five Ca^2+^-binding EF-hands) are shown. The catalytic triad residues are boxed, highlighted in green and marked with a star underneath (C:cysteine; H: histidine, N: asparagine).
(DOCX)

Figure S3 **Comparison of the deduced amino acid sequences of Clpn3 of catfish and other organisms.** Comparison of the deduced amino acid sequences of the catfish Clpn3 with homologous sequences from *Danio rerio* (Dre, GenBank, AAH81672), *Salmo salar* (Ssa, GenBank, NP001158880) and *Hippoglossus hippoglossus* (Hhi, GenBank, ACY78226). Identical amino acid residues have been highlighted. The four catalytic subunits: I (pro-peptide), II (Cysteine catalytic site), III (“electrostatic switch”), and IV (five Ca^2+^-binding EF-hands) are shown. The catalytic triad residues are boxed, highlighted in green, and marked with a star underneath (C: cysteine; H: histidine, N: asparagine). PEST proteolytic signals indicating target protein for rapid destruction are highlighted in yellow, and are marked underneath.
(DOCX)

Table S1 **List of primers used in qRT-PCR for expression analysis of** ***clpn1, clpn2, clpn3*** **, and titin genes of channel catfish.** β-actin gene was used as internal control.
(DOCX)
