# Supplementary material for: Effect of Nutrient Restriction and Re-Feeding on Calpain Family Genes in Skeletal Muscle of Channel Catfish (Ictalurus punctatus)
Source: PLoS One. 2013 Mar 19;8(3):e59404. doi: 10.1371/journal.pone.0059404 (PMC3602173; doi:10.1371/journal.pone.0059404)
Supplement: Figure S1 — Comparison of the deduced amino acid sequences of calpain 1 orthologs in catfish and other organisms. Comparison of the deduced amino acid sequences of catfish calpain 1 with homologous sequences of Danio rerio (Dre, GenBank, AAF82808), Oncorhynchus mykiss (Omy, GenBank, AAT81416), and Hippoglossus hippoglossus (Hhi, GenBank, ACY78224). Identical amino acid residues are highlighted. The four catalytic subunits: I (pro-peptide), II (Cysteine catalytic site), III (“electrostatic switch”), and IV (five Ca2+-binding EF-hands), are shown. The catalytic triad residues are boxed, highlighted in green and marked with a star underneath (C: cysteine; H: histidine, N: asparagine). (DOCX) [file pone.0059404.s001.docx]

**Figure S1**

Domain I

Ipu --MEPIY-ATGVAAKLRSQWDRNEGLGQNYNAVKFLGQDYEMLRAQCLQSRTLFEDSYFPATASSLGFNELGPRSSKTSGVRWMRPPEICSRPQFITQGATRTDICQGALGDCWLLAAIA 117

Omy --MEQVY-ASGMAAKLRSQWDRDEGLGQNHNAVKFQGQDFKSLRARCLQSRSLFEDSLFPCASSSLGFNELGPRSSKTQGVRWMRPTEICTRPQFIVDGATRTDICQGALGDCWLLAAIA 117

Hhi MGEERIYTATGMAARLRSQWDRDEGLGQNDNAVKFLGQDYESLRAQSLQSRRLFEDNLFACASSSLGFDELGPKSSKTQGVRWMRPTEFCKRPEFIVDGATRTDICQGALGDCWLLAAIA 120

Dre --MEPIC-ATGMAARLRSQWDRDAGLGQNHNAVKFLGQDYETLRAQSQQSRRLFEDPMFTASSSSLGFNELGPRSSKTQGVRWMRPKEMCARPQFIVDGATRTDICQGALGDCWLLAAIA 117

*

Domain II

Ipu SLTLNDNLLHRVVPHGQSFSEGYAGIFHFQFWQFGEWVDVVIDDRLPFKDGKLLFVHSAEGGEFWSALVEKAYAKLNGCYEALSGGSTSEGFEDFTGGVTEMYELSKPPADLYTIIKRAV 237

Omy SLTLNDNLLHRVVPHGQDFRGQYAGIFHFQFWQYGEWVEVVIDDRLPVKDGKLLFVHSAEGGEFWSALLEKAYAKLHGCYEALSGGSTSEGFEDFTGGVTEMYELRKAPSDLYSIISRAV 237

Hhi SLTLNNSLLHRVVPHGQGFQQGYAGIFHFQFWQFGEWVEVVIDDRLPVKDGKLLFVHSVEGTEFWSALLEKAYAKLNGCYEALSGGSTSEGFEDFTGGVTEMFDLAKAPSDLYSIIKRAI 240

Dre SLTLNDNLLHRVCRTGQDFDSRYAGIFHFQFWQFGEWVDVVIDDRLPTKDGKLLFVHSAEGGEFWSALLEKAYAKLNGCYEALSGGSTCEGFEDFTGGVTEMYELKKAPADLFSIIGRAI 237

Ipu ERGSLLGCSIDISNTRDMEAVTFKKLVKGHAYSVTGVEEVNFRGTPTKLVRIRNPWGEVEWTGAWSDNSREWESVDRSVRGRLQNRSEDGEFWMSFSDFLREFTRLEICNLTADALEANQ 357

Omy ERGSLLGCSIDITGSQDMEAVTFKKLVKGHAYSVTGVDEVVYRGNMTKLVRIRNPWGEIEWTGAWSDNSREWDGVDRSVRGRLQNQSEDGEFWMSFSDFLREFSRLEICNITADALQNSQ 360

Hhi ERGSLLGCSIDITSTRDMEAVTFKKLVKGHAYSVTAVDEVVYRGNMTKLVRIRNPWGEVEWTGAWSDNSREWDNVDRSVRGRLQNRSEDGEFWMSFSDFLREFTRLEICNLTADALQASQ 357

Dre ERGSLLGCSIDITSKFDMEAVTFKKLVKGHAYSVTGAEEVVYRGNMTKLVRIRNPWGEVEWTGAWSDNAREWDSVDPSTRSKLNNRSEDGEFWMSFQDFLREYSRLEICNLTADALEASQ 357

*

*

Domain III

Ipu IKKWSTSLYQGEWRRGSTAGGCRNYPATFWINPQFKINLKIPDSAGKNDCTFLVALMQKDRRKQRKEGKDMETIGFAVYEVPSEFVGQPAVHLKRDYFITHGSSARSETFINLREVSSRL 477

Omy QKKWSSAVYQGEWRRGSTAGGCRNFPATFWINPQFKIDLQHPDTAGQSDCSFLVALMQKDRRKKRKEGKDMETIGFAIYEVPNEYVGRSGIHLKRDFFLTHGSSARSELFINLREVSSRF 477

Hhi LKKWSSSLYQGEWRRGSTAGGCRNFPATFWLNPQFKIALQHPDAPGQSECSFLVALMQKDRRKKRREGKDMETIGFALYEVPDEFAGRSGVHLKRDFFLTHGSSARSELFINLREVSSRL 480

Dre VKKWSTANYNGEWRRGSTAGGCRNYPATFWINPQFKVALKNPDSPGQSECSFLVALMQKDRRKKRREGQDMETIGFAIYEVPREFLGQSGVHLKRDFFLTHASSARSELFINLREVSSRF 477

Ipu RLPVGEYIIVPSTFEPQKEANFVLRVFSEKPADSVELDDEVKADLPKVNYLAESQIDAGFKGLFRQLAGADMEISVTELQTILNRIISKHKDLKTDGFTQEACRSMINLMDVDGSGKLGL 597

Omy QLPAGEYIIVPSTFEPQKEGDFVLRVFSEKPANSEELDDDVTAELPAESQLDESQIDAGFKSLFRQLAGEDMEISATELQTILNRIISKHKDLKTDGFGPEACRTMINLMDTDGSGKLGL 597

Hhi RPPAGEYIIVPSTYDPNKEADFVLRVFSEKPAASEELDDKVEAELPTEIELDESQIDAAFKNLFRQLAGPDMEISLTELQTILNRIISKHKDLKTDGFTKEACRSMINLMDTDGSGKLGL 600

Dre RLPAGEYIIVPSTFEPNKEADFVLRVFSEKPANSEEMDDKVMAEIPEEQRLDESQIDAGFKSLFRQLAGADMEISVTELQTILNRIIAKHKDLKTDGFGKESCRSMINLMDTDGSGKLGL 597

Domain IV

Ipu TEFHVLWEKIKQYLEVFRKFDVDKSGTMSSYELRMALESAGIKLNNHIFQLIILRYAEQDLTVDFDNFVCCLVRLETMFKTFYTLDTDTDGIISLDFNQWLSLTMFV 704

Omy AEFHVLWEKIKRYLTVFRQFDLDKSGTMSSYEMRMALEAAGFKLKNHLFQLIILRYTEADLTVDFDNFVTCLVRLETMFKTFKTMDADSDGVIELNFFQWITLTMFA 704

Hhi TEFHVLWEKVKRYLTIFRQFDLDKSGTMSSYEMRMALESAGFKLTNHLFQLIILRYTEADMAVDFDNFVTCLVRLETMFKTFKTLDTDADGQITLNFYQWITLTMFA 707

Dre VEFHVLWEKIKRYLQIFRDHDVDKSGTMSSYEMRKALETAGFKLNNHLFQLIILRYTEEDLSVDFDNFVSCLVRLETMFKTFKSLDTDADGVISLTFFQWITLTMFA 704
