# Supplementary material for: Effect of Nutrient Restriction and Re-Feeding on Calpain Family Genes in Skeletal Muscle of Channel Catfish (Ictalurus punctatus)
Source: PLoS One. 2013 Mar 19;8(3):e59404. doi: 10.1371/journal.pone.0059404 (PMC3602173; doi:10.1371/journal.pone.0059404)
Supplement: Figure S2 — Comparison of the deduced amino acid sequences of Clpn2 of catfish and other organisms. Comparison of the deduced amino acid sequences of catfish Clpn2 with homologous sequences of Danio rerio (GenBank, NP_001017807), Oncorhynchus mykiss (GenBank, NP001117963) and Hippoglossus hippoglossus (GenBank, ACY78225). Identical amino acid residues have been highlighted. The four catalytic subunits: I (pro-peptide), II (Cysteine catalytic site), III (“electrostatic switch”), and IV (five Ca2+-binding EF-hands) are shown. The catalytic triad residues are boxed, highlighted in green and marked with a star underneath (C:cysteine; H: histidine, N: asparagine). (DOCX) [file pone.0059404.s002.docx]

**Figure S2**

Ipu MSGVASTLAKKRARAAGFGTNANANKYLNQDYEALRSQHQG--QLFCDPYFPAAPESLGFQELGPNSSKTRGVQWKRPGELCSRPQFIVGGATRTDICQGALGDCWLLAAIASLTLNQDV 118

Domain I

Omy MAGVASTLAKKRALAAGFGTNANASKYLNQDFESLRSECLGRGSLFTDSTFPAEPASLGFNELGPRSSKTRGVQWKRPGELVSSPEFIVGGASRTDICQGGLGDCWLLAAIASLTLNEDV 120

Hhi MAGVASTLAKQRALAAGFGTNANAVRYLNQDFGSLRAKCRSAGRLFCDPTFAAAPESLGFKELGQSSHKTRGVTWKRPTELVSNPEFILGGATRTDICQGALGDCWLLAAIASLTLNEYV 120

Dre MSGIATKLQKNRARAAGIGTNAQAAKFLNQDYEALKRECLESGRLFQDGMFEANVSALGFKELGPNSSKVRGVEWLRPKQLTSNPTFISGGATRTDICQGALGDCWLLAAIASLTLNQDV 120

*

Domain II

Ipu MARVIPSGQDFE-SGYAGIFHFQFWQFGEWVDVVIDDRLPTKDGKLLFVHSAEGNEFWSALLEKAYAKANGSYEALSGGSTTEGFEDFTGGIAEQYELRNAPANMFQIIQKALASGALLG 237

Omy MARVVPEGQGFGRGEYAGIFHFQFWQFGEWVDVVIDDRLPTKDGKLLFVHSAEGSEFWSALLEKAYAKVNGCYEALSGGSTTEGFEDFTGGIAENYDLKKAPSDLFQIIRKALASGALLG 240

Hhi MARVVPTDQGFG-DDYAGIFHFQFWQYGEWVDVVIDDRLPVKDGELMFVHSAEGREFWSALLEKAYAKVNGCYEALSGGSTTEGFEDFTGGIAENYDLKQPPSNLFQIIKKSLEAGALLG 239

Dre FARVVPAGQSFD-GDYAGIFHFQFWQFGEWVDVVIDDRLPARKGELLFVHSAEGSEFWSALLEKAYAKLNGCYEALSGGTTTEGFEDFTGGIAEVHELPKAGPNLFKTIQKALSWGSLLG 239

Ipu CSIDITSAADSEAITRQKLVKGHAYSLTGAVEVNYRGSLEKLVRMRNPWGQVEWTGAWSDGSCEWNSVDPS--ERPNANAEDGEFWMSFSEFQRQYSRIEICTLTPDA--ITSDQVKPWS 353

Omy CSIDITSQADSEAVTYQKLVKGHAYSLTGAMEVNYRGRKEKLVRVRNPWGTVEWTGAWSDNSSEWNSVDFS--ERDVVKADDGEFWMSYTDFMKNYHRLEICTLTPDTLLLTTDDVKHWS 358

Hhi CSIDITSAADSEAVTRQKLVKGHAYSLTGAVEVNYRGRQEKLVRIRNPWGQVEWTGAWSDGSSEWNSVQG---DCPNANAEDGEFWMSYSDFLRHYSRIEVCTLTPDT--IEDDSVKHWS 354

Dre CSIDITSSSDSEAITSQKLVKGHAYSVTGAEEVEYRGDLTKLIRIRNPWGQVEWTGPWSDGSSEWRQISDSDRERLSSKAEDGEFWMSFSDFMRHYSRVEICNLTPDA--LTDESVNKWA 357

*

*

Domain III

Ipu VKNYSGNWRRGSTAGGCRNHAQTFWMNPQFVIKVNEEDDDPNDNEKGCSLLVGLIQKNRRRLRKEGGDMHTIGYAIYEVPSQFQGQTNLHLDKNFFLTHAQTARSETFINLREVSTRFKL 473

Omy VSNYDGAWRKGSTAGGCRNNPYTFWMNPQFKIKLEEEDDDPGDDEVGCSFVVGLIQKNRRRMRKAGEDMHTIGFAIYEVPEQFHGQREVHLDKNYFLSHAQTARSETFINLREVSTRFKL 478

Hhi VSTFDGTWRRGSTAGGCRNNPYTFWTNPQFVIKLDEEDDDPDDGEVGCSFVVGLIQKNRRKLRKQGEDMHTIGFAIYEVPKQFQGQREVHLDKNFFLSHAQTAKSETFINLREVSSRFKM 474

Dre LSKFDGNWRNGSTAGGCRNYPNSFWMNPQFLIKLEEQDDDPTDNEAGCSFVVGLIQKNRRKMRKVGEDMNTIGFAIYEVPDEFVGQRNVHLDRNFFVRHASAARSETFINLREVCSRFCL 477

Ipu PAGEYLVVPSTFDPHLDGDFCLRVFSEKQTETQRCDDPVDAKLEDETVSDSEVEGSFRSLFMKLAGADMEISAVELRSILNKVVAKRTDIKTDGFSLDTARTMVNIMDDSGNGKLGLGEF 593

Omy PPGEYLIVPSTFEANLNGDFCLRVFSEKQAETLPCDDPVKAELEDDTVPEGEVDAGFRGLFTKLAGDDMEISASELRSIFNKIVAKRTDIKTDGFSLDTARIMVNLMDDSGNGKLGLVEF 598

Hhi PPGEYLIVPSTFEAHLNGDFCIRVFSEKQSETQPCDDPVEAELEDETVSDEEVDAGFRGLFSKLAGDDMEISAVELKTIMNKIVGKRTDIKTDGFSMETCRVMVNLMDDSGNGKLGLGEF 594

Dre PPGEYLIVPSTFEPNKDGDFCVRVFSEKQAEFQELDDPVESKVAEIEIKEGDIDSRFRNLFKQLAGADSEISAFELQKILNNVIAKRKDIKTDGFSLETCRNMVNLLDKDGTGKLGLLEF 597

Domain IV

Ipu ATLWKKVQRYMNIYKENDMDNSGNISTTELRGALAKAGFSLNDTIFQLLVARYAVTDLTIDFDDFVGCLMRLELMFRVFRKMDPHNTGFLEMDFQMWLCLTMI 696

Omy ATLWKKIQKYLSIYKSNDMDGSGCMSTPEMRMALNKAGFSLNNTLHQVLAARYGEADMTIDFDNFVACVMRLEMMFKVFKKLDMDDTGFIELDFFQWLSFSMI 701

Hhi ATLWKKVQRYLSIYKKNDSDNSGTMSTPEMRVAFKDAGFSLNNTIYQQLVARYSDPDMTIDFDNFVGCLMRLEMMFKIFKKLDAHNSGSIELDFNQWINFAMI 697

Dre KILWTKIELFVDVYSKNDKDQSGTMSSMEMREAVEKAGFSLNNALHQILVARYSEPNLTIDFDNFVACLIRLECMFKAFKVLDKDKNGTVELNMMEWLNVSML 700
