# Supplementary material for: Effect of Nutrient Restriction and Re-Feeding on Calpain Family Genes in Skeletal Muscle of Channel Catfish (Ictalurus punctatus)
Source: PLoS One. 2013 Mar 19;8(3):e59404. doi: 10.1371/journal.pone.0059404 (PMC3602173; doi:10.1371/journal.pone.0059404)
Supplement: Figure S3 — Comparison of the deduced amino acid sequences of Clpn3 of catfish and other organisms. Comparison of the deduced amino acid sequences of the catfish Clpn3 with homologous sequences from Danio rerio (Dre, GenBank, AAH81672), Salmo salar (Ssa, GenBank, NP001158880) and Hippoglossus hippoglossus (Hhi, GenBank, ACY78226). Identical amino acid residues have been highlighted. The four catalytic subunits: I (pro-peptide), II (Cysteine catalytic site), III (“electrostatic switch”), and IV (five Ca2+-binding EF-hands) are shown. The catalytic triad residues are boxed, highlighted in green, and marked with a star underneath (C: cysteine; H: histidine, N: asparagine). PEST proteolytic signals indicating target protein for rapid destruction are highlighted in yellow, and are marked underneath. (DOCX) [file pone.0059404.s003.docx]

**Figure S3**

Domain I

Ipu MKKSKISKTSEKEKREVQETVSETRARTTEAASGAPAGVFQPPGNLYSAILSRNQAVKDAKRLKDFLELREKYVRKKVLFKDPLFPADDSSLYYSQK-----FPLNLVWKRPSEICDKPQ 115

Ssa ----------MSLVEETSVKVALETEASPDVPEGKTDYPFTASNSIYTAILSRNEAVKDAKRLKNFLELRDKYVHKKVLFEDPLFPPNDSSLWYSSK-----FPIEFEWKRPTEICDNPQ 105

Hhi -MEDKTHEGKVPLVEDTQVKVLHETEAS-HGPDDKADYPPVGANSIYSAILSRNEAVKDAKRLKTFLELRDKYLKKKVLFEDPLFPANDSSLFYSRK-----PSMKIEWKRPSEICDKPE 113

Dre ----------MPYTPSGFFCDRLIRERERRDGEGSLSKPIRFSG-------------------QDYTLLKQEYLQKKTLFEDETFPATVDSLGYKELGHKSNKVKNIVWKRPKEICDNPQ 91

Ipu FIIGGANRMDICQGDLGDCWLLAAIACLTLNETLRYRVVPPDQSFTENYAGIFHFQFWRYGEWVDVLVDDRLPTYKNRLVFTRSGNKNEFWSALLEKAYAKLHGSYEALKGGNTLEAMED 235

Ssa FIIDGASRTDICQGELGDCWLLAAIACLTLNDKLLYRVIPPDQSFTENYAGIFHFQFWRYGEWVDVVVDDRIPTSNNQLVFTKSFRKNEFWSALLEKAYAKLHGSYEALKGGNTLEAMED 225

Hhi FIVDGANRTDICQGELGDCWLLAAIACLTLNEKLLYRVIPPDQSFTDNYAGIFHFQFWRYGEWIDVVVDDRIPTCNNQLVFTKSFRKNEFWSALLEKAYAKLHSSYEALKGGNTLEAMED 233

Dre FIVGGASRTDICQGDLGDCWLLAAIACLTLNDKLLYRVIPQEQSFSEQYAGIFHFQFWRYGDWVDVVVDDRIPTFNNQLVFTKSAERNEFWSALLEKAYAKLHGSYEALKGGNTAEGMED 211

*

Domain II

Ipu FTGGVTEYYEITDAPKEIYNIMRKALERGSLMGCSIDALVPTASETKTSTGLVRGHAYSVTGVEQGKQ-QDGRDTRIRLVRIRDPWGVAP---PPSCKSNDWTKLVTSEQEKQKLQPVGP 351

Ssa FTGGVTEFFEMSEAPKELYKIMKKALERGSLMGCSIDALLPSHCETETANGLVRGHAYSITALEECDKLKVTKDTKIRLVQMRNPWGMVLWKGPWNAKSKEWSTISSSDKARLKKKTVET 345

Hhi FTGGLTEFFELSAASKDLYNIMKKALERGSLMGCSIDVVSASEMETRTDHGLVKGHAYSIIGLEECD--EVAKDTKIRLIRLRNPWGWVLWKGPWSADSKEWSTISIADKENLKKQTVEA 351

Dre FTGGVTEFYEMKEAPKELYKIMQKALERGSLMGCSIDSLVPARFETRTATGLVKGHAYSVTAVEECKQ-SQQKESRVRLVRLRNPWGQVEWNGPWSDNSKEWESLSKAEKEKLQQQNAED 330

*

*

Ipu GEFWMYFEEFQKTFTKLEICNLTPDALQDDELLKWTVSVNEGRWVRGCSAGGCRNYPDTFWTNPQYRLRLTEEDDDEPEYGEKGCTVVVALMQKGRRRESYAGATLHAVGFAIYEVPKEM 471

Ssa SETWISFEDFKKNFTKLEICNLTPDTLLDDKSHSWTVAVNEGRWVRGSSAGGCRNFPDTFWTNPQYRMRLYEEDDDPDDPEHVACTVMVALMQKGRRKDRSKGAKLYTIGFSIYEVPKEM 465

Hhi SEFWMSFDDFKRNFTKLEMCNLTPDALQGDERNTWTVSVNEGRWVRGSSAGGCRNFPDTFWTNPQYRLQLYEEDDDPED-GTVACTLVVALMQKGRRMQRHQGAKFLTIGFSIYEVPKEM 470

Dre GEFWMSFEDFKKNYTKIEICNLTPDALEDDKLHKWTVSVNEGRWVRGCSAGGCRNYPDTFWTNPQYRLRLLEEDDDPED-DEVACTFVVALMQKNRRKERKLGANLLTIGFSIYEVPKEM 449

PEST sequence

Ipu HGNKQHLPKDFFLYNASKARCKSYINLREVTERFCLSPGEYVIVPSTFEPHKESDFLLRVFSEKKSTSELMGAVIEAEPYLLENG----------------------------------K 557

Ssa IGSQQHLQKDFFLYNASKAKCKSYINLREVSERFCLPPGEYVIIPSTFDPHEEGEFILRVFSEKNSTSEETENTITSDQIEQGKNRTKKGKPIVFVSDRAKANKEIEQDDIEVQKEKKEK 585

Hhi CGQNQHLQKDFFLYTASKAKCKTYINLREVTERFRLPPGEYAILPTTFEAHQEGEFLLRVFSEKQSTSEEAENTIESGKIQQDKR--KREKPIVFVSDRARANKEIEHDGIRG--EKKKK 586

Dre HGNKQHMQKEFFMSTTAKARSRAYINLREVTQRFRLSPGEYVIIPSSYEPHQEGEFILRVFSEKRNTSEEIENRIEAD-----------------------------------------H 528

Domain IV

Domain III

Ipu NKIKLFE-EEESEEEKQFRAVFEQIS------------GDDMQINAKELTIVLNKAVSKYKELKS-EGFSLDSCRSMIALMDTDGTGRLNLQEFKHLWNKIKQWKIIFTRYDTDKSGTIS 663

Ssa VKPKPQQPDEESGEHKMFRAIYQQIA------------GEDMQICANELKVVMKRVLEKHNEMKT-DGFSLESCRSMIALMDTDGTGKLNLQEFKHLWSKIKAWQLIFKKYDKDKSSTIS 692

Hhi PKRKLLEPEEETEEEKQFRAIYQQIS------------GEDMQICANELQTIMKNVLSKHGEIKTKEGFSLETCRSMIALMDTDGTGKLNLQEFKHLWKKIKKWQMIFKRYDKDKTCSIS 694

Dre PVPSPASTGEESEEDQQFLSIFQHIAGDVSTGEAVSAWGSNMEVSANELKDVLNKVVSKHKDIHT-ESFSRECCRSMIALMDMDGTGRLNLQEFRQLWNKIKQWQEIFKRYDFDHNDTIS 647

Ipu SFEMRNAVADAGFQLNPQLHDIIAMRYANEHLYLDFDSYICCLVRLEGMFRAFKAFDNDGDGLIKLNVLEWLQLTMYA 741

Ssa SFEMRNAVNDAGFHLNNQLYDIITMRYADEHLNIDFDSFICCFVRLEGMFRTFHAFDKNGDGTIKLNVLEWLQLTMYA 770

Hhi SFEMRNTVNDAGFHLNKQLYDIIAMRYADEHLNIDFDSYICCFVRLEGMFRAFNAFDKDGDGIIKLNVLEWLQLTMYS 772

Dre SYEMRNAINDAGFRLNNQLYDIITMRYANESMNVDFESFISCLVRLEGMFRAFQAFDQCGDGSIRLSVLEWLQLTMYA 725
