# Supplementary figures and images for: Effect of Nutrient Restriction and Re-Feeding on Calpain Family Genes in Skeletal Muscle of Channel Catfish (Ictalurus punctatus)
Source: PLoS One. 2013 Mar 19;8(3):e59404. doi: 10.1371/journal.pone.0059404 (PMC3602173; doi:10.1371/journal.pone.0059404)

**Table S1**


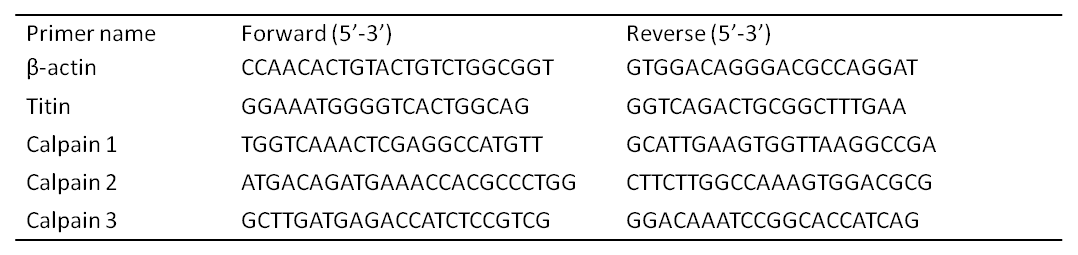

Supplement: Table S1 — List of primers used in qRT-PCR for expression analysis of clpn1, clpn2, clpn3 , and titin genes of channel catfish. β-actin gene was used as internal control. (DOCX) [file pone.0059404.s004.docx]
